# Targeted parallel sequencing of large genetically-defined genomic regions for identifying mutations in *Arabidopsis*

**DOI:** 10.1186/1746-4811-8-12

**Published:** 2012-03-30

**Authors:** Kun-hsiang Liu, Matthew McCormack, Jen Sheen

**Affiliations:** 1 Department of Molecular Biology and Center for Computational and Integrative Biology, Massachusetts General Hospital, Boston, MA 02114, USA; 2 Department of Genetics, Harvard Medical School, Boston, MA 02114, USA

**Keywords:** Next generation sequencing, EMS, PCR-amplified genomic library, Nitrate signalling, Positional cloning

## Abstract

Large-scale genetic screens in *Arabidopsis *are a powerful approach for molecular dissection of complex signaling networks. However, map-based cloning can be time-consuming or even hampered due to low chromosomal recombination. Current strategies using next generation sequencing for molecular identification of mutations require whole genome sequencing and advanced computational devises and skills, which are not readily accessible or affordable to every laboratory. We have developed a streamlined method using parallel massive sequencing for mutant identification in which only targeted regions are sequenced. This targeted parallel sequencing (TPSeq) method is more cost-effective, straightforward enough to be easily done without specialized bioinformatics expertise, and reliable for identifying multiple mutations simultaneously. Here, we demonstrate its use by identifying three novel nitrate-signaling mutants in *Arabidopsis*.

## Background

Genetic screens are a powerful approach for studying diverse processes by isolating mutants showing phenotypes directly or indirectly involved in biological pathways. Identifying the molecular lesion underlying these phenotypes is crucial towards understanding the mechanism of the process it is involved in. In order to reveal the molecular identity of the mutant, positional cloning is commonly employed to identify the mutations [1]. However, despite the availability of the *Arabidopsis *genome sequence, positional cloning from diverse mutant screens can be time-consuming or even hampered due to low chromosomal recombination in megabase-sized regions surrounding the mutation [1-4].

Next-generation sequencing (NGS) technology for whole-genome sequencing (WGS) provides an alternative method for molecular characterization of mutations [5]. However, the copious numbers of mutations generated during the mutagenesis processes become a hindrance due to the presence of hundreds or thousands of mutations unrelated to the specific phenotype. This introduces a high degree of complexity in subsequent WGS data analysis aimed at identifying mutations responsible for the phenotypes. Specialized computational methods, hardware, and expertise, not available in most laboratories, are typically needed to accomplish the analysis. Backcrosses mutants to wild type plants for several generations can attenuate complexity by eliminating unrelated mutations [6], but this is very time consuming when using *Arabidopsis*. Improved approaches, SHOREmap and Next-Gen Mapping (NGM), combine integrated mapping with NGS and have led to identification of EMS (ethyl methanesulfonate)-generated mutation sites in *Arabidopsis *[7-9]. However, these strategies require whole genome sequencing, and so huge amounts of uninformative non-target regions are sequenced which is very costly and can be impractical for many laboratories involved in genetic studies. For example, based on published reports, characterizing one mutant in *Arabidopsis *usually takes one flow cell (7-8 lanes) using paired-end reads of 38-40 cycles [7-9]. The possibility of using only one lane of a flow cell and a few F2 lines to identify mutations in a single mutant is described in one report [7]. However, detection of the known mutations were found only in some cases using one-lane sequencing due to variable and low coverage of the genome [7].

It is both costly and time consuming to associate a single mutant phenotype with its underlying molecular mutation. The ability to simultaneously characterize multiple mutants reduces both cost and labor, and greatly accelerates the association of genes with pathways. Recognizing the benefits of characterizing a large number of mutants at a molecular level in order to dissect complex signaling networks, and also being aware of current technical and financial limitations, we have created a streamlined method, targeted parallel sequencing (TPSeq), for efficient and simultaneous identification of multiple causative mutations in *Arabidopsis *and other genetic model organisms. The method requires only simple and quick mutant mapping using polymerase chain reaction (PCR) markers accessible to every laboratory [1-4]. We have used this method to simultaneously identify three novel nitrate-signaling mutants with altered nitrate marker gene responses and nitrate-based growth phenotypes.

## Results and discussion

### Isolation of nitrate signaling mutants by a dual-screen

Nitrate is central to plant gene regulation and growth. However, little is known about the molecular mechanisms of nitrate signaling and also the genetic basis of diverse nitrate-associated traits in plant growth and development. Currently, a few transcription factors, protein kinases, microRNAs and a transporter-sensor have been reported to participate in regulating nitrate-responsive gene expression and growth in a context dependent manner [10-13]. Discovery of new signaling components and the connection of existing regulatory nodes in the nitrate-signaling network remain challenging.

Forward genetic screen is a very powerful approach as an initial analysis aimed at identifying novel signaling components. We designed a dual genetic screen strategy to isolate mutants involved in nitrate signaling. We first screened for mutants having a deregulated nitrate responsive gene expression pattern. We selected nitrite reductase (*NIR*) as our nitrate response marker gene because *NIR *plays a critical role in the nitrate assimilation pathway, it is encoded by a single gene, and *NIR *expression can be rapidly and consistently induced by nitrate [14]. In order to monitor nitrate responses, we generated an *Arabidopsis *transgenic line harboring a nitrate responsive luciferase (LUC) reporter driven by the *NIR *gene promoter. In the first screen, two classes of mutants were isolated by measuring LUC activities in a 96-well plate assay. EMS-mutagenized seeds were placed in a 96-well plate and LUC activities were measured with a scintillation counter. The *nitrate insensitive *(*nis*) mutant showed reduced LUC activity after nitrate induction, whereas the *nitrate constitutive response *(*ncr*) mutant exhibited higher LUC activity in the absence of nitrate induction. Approximately 25,000 M2 seedlings were screened. A total of 273 *nis *mutants and 65 *ncr *mutants were isolated during the first step of the screen. Of these, 4 *nis *and 5 *ncr *mutants were further confirmed in the second generation.

**Figure 1:**
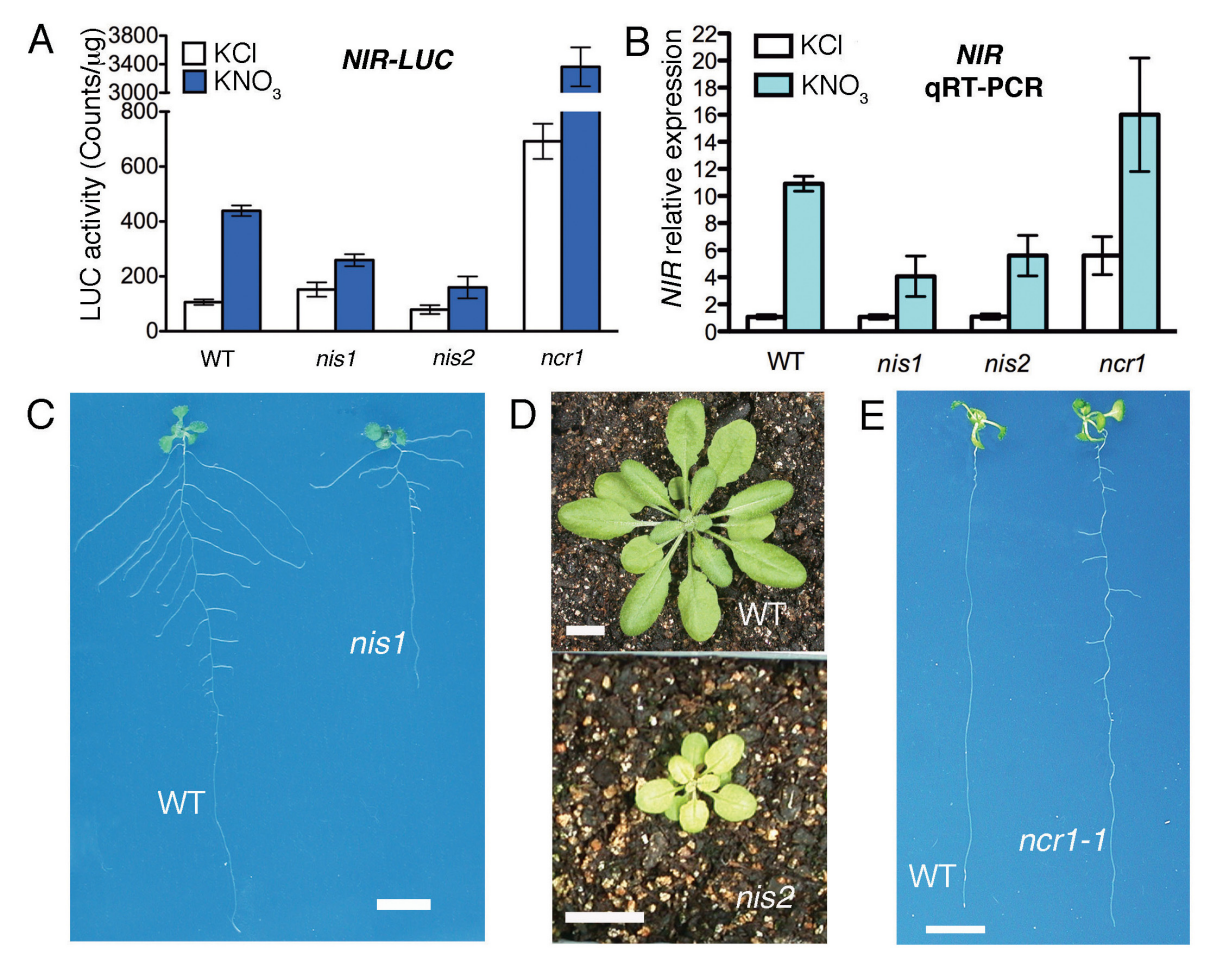
**Phenotypic analysis of *nis1, nis2, ncr1-1***. **A**. Comparison of *NIR-LUC *activity in *nis1, nis2 *and *ncr1-1*. LUC activities were measured after 2 h incubation with either 10 mM KCl or KNO_3_. The *NIR-LUC *transgenic line in Col is used as the wild type control. Three seedlings were pooled and grinded for protein concentration determination and LUC activity analysis. Values shown are means ± s.d. of three or four biological replicates. **B**. Relative endogenous *NIR *expression in *nis1, nis2 *and *ncr *as measured by real-time PCR. Plants were treated with either 10 mM KNO_3 _or KCl for 2 h. Relative expression of *NIR *is normalized to the expression of *TUB4*. The relative expression level is calculated relative to the value of wild type treated with KCl. Values shown are means ± s.d. of three biological replicates. **C**. Altered root architecture in *nis1*. Plants were grown on medium containing 2.5 mM ammonium succinate for 3 days and transferred to medium containing 5 mM KNO_3 _for 8 days. **D**. *nis2 *showing small pale-green leaves after plants grown in soil for 33 days. **E**. The lateral root de-suppression phenotype in *ncr1-1*. Seedlings were grown on medium containing 50 mM KNO_3 _as the sole nitrogen source for 14 days. Scale bar = 1 cm.

As alternations in a nitrate-responsive marker gene may or may not be linked to complex nitrate-associated growth phenotypes, we performed a secondary screen with *nis *and *ncr *mutants based on well-known nitrate-associated traits. We conducted three distinct assays, including nitrate (5 mM) promotion of lateral root growth, high nitrate (50 mM) inhibition of lateral root emergence, and nitrate-associated greening and leaf expansion. This second screen yielded three mutants, *nis1, nis2 *and *ncr1*, with reproducibly altered *NIR-LUC *expression patterns (Figure 1A) and nitrate-associated traits in the next generation. We further confirmed by reverse transcriptase-quantitative PCR (qRT-PCR) that the endogenous *NIR *gene expression displayed similar changes in nitrate responses as the *NIR-LUC *transgene in *nis1, nis2 *and *ncr1*, respectively (Figure 1B). The *nis1, nis2 *and *ncr1 *mutants represented new classes of nitrate signaling mutants as they displayed nitrate-specific response alternations in *NIR *promoter and transcript regulation, which are not influenced by other nitrogen sources, including ammonium or glutamine (Figure 1A and 1B, and data not shown). Unexpectedly, these mutants exhibit distinct nitrate-associated traits in secondary screens: *nis1 *is deficient in nitrate-promoted root growth (Figure 1C), *nis2 *has small pale green leaves (Figure 1D), whereas *ncr1 *lacks high-nitrate inhibition of lateral root elongation (Figure 1E). The mutant phenotypes of *nis1 *and *ncr1 *are observed only on nitrate medium, but the phenotype of *nis2 *persists in medium with different nitrogen sources (Figure 1 and data not shown).

### Identification of mutation sites by TPSeq

**Figure 2:**
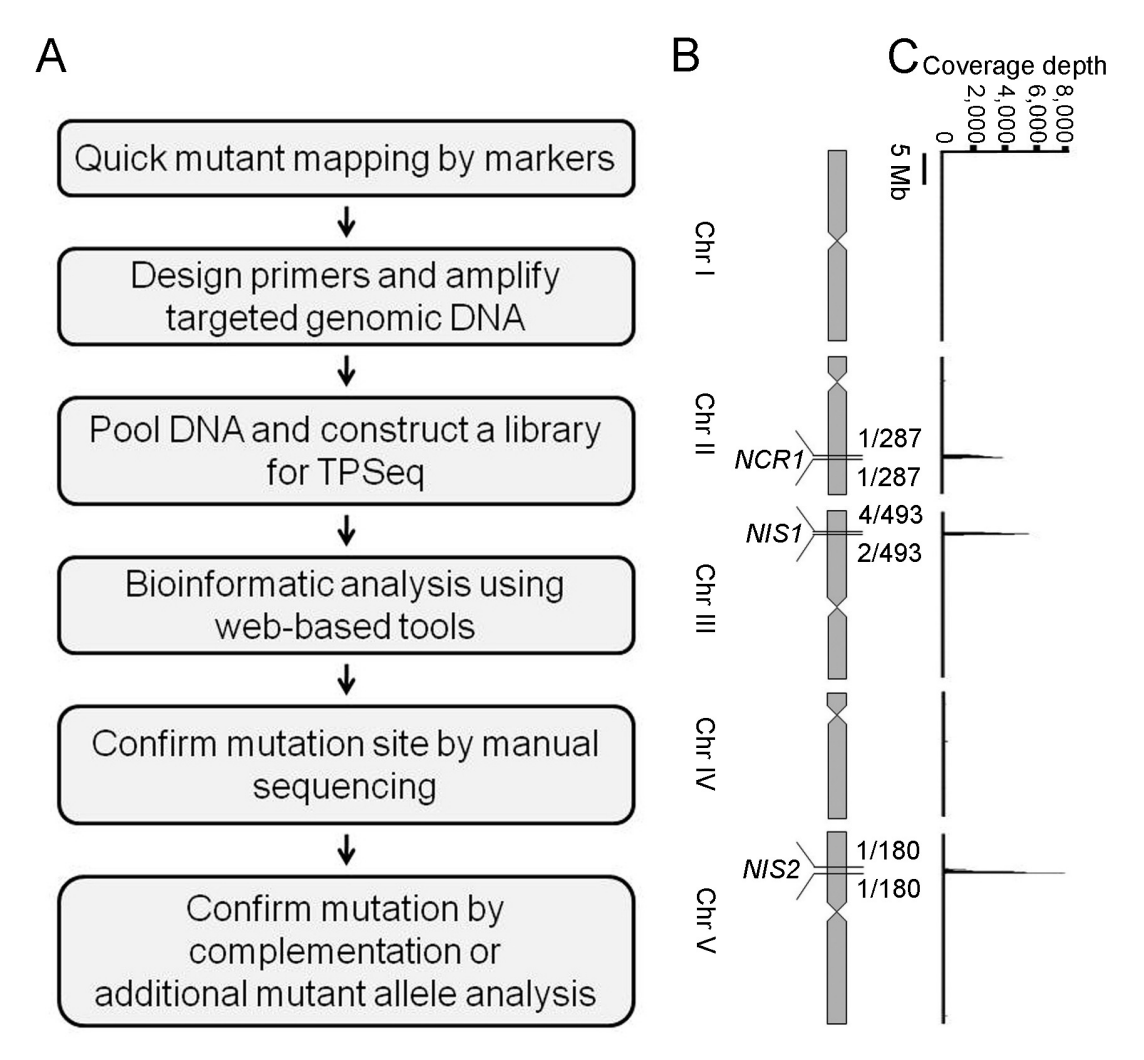
**Identifying mutations by TPSeq**. **A**. Flowchart of the TPSeq procedure. **B**. Physical map of mutations on *Arabidopsis *chromosomes. Three mutants were mapped to different chromosomes with the numbers of recombinants and nearest markers. **C**. Coverage plot from TPSeq. Y-axis is the average read of 100 kb window. X-axis is the corresponding location on chromosome shown in **B**

Moving toward a molecular understanding of nitrate signaling, it is necessary to reveal the molecular identity of *NIS *and *NCR *genes. We have developed an efficient and low-cost strategy, TPSeq, to simultaneously identify multiple genetic mutations in *Arabidopsis *(Figure 2A). *Arabidopsis *has long been used for genetic studies and the entire genome was sequenced ten years ago. There are many available molecular markers based on sequence polymorphism among *Arabidopsis *accessions, which allow for quick mapping to narrow down mutations in relatively much smaller target regions [1-4]. Quick mapping was performed by taking advantage of simple PCR-based methods using simple sequence length polymorphism (SSLP) or cleaved amplified polymorphic sequences (CAPs) markers [1]. After quick mapping, *NCR1 *was located in the interval between 13.89 Mb and 14.43 Mb on Chromosome II by isolating 287 independent recombinants. *NIS1 *was mapped to the upper arm of Chromosome III between 2.82 Mb and 3.23 Mb by isolating 493 independent recombinants, and *NIS2 *was mapped to the upper arm of Chromosome V between 4.66 Mb and 5.39 Mb by isolating 180 independent recombinants (Figure 2B). All three mutants were recessive. The phenotypes of the mutants co-segregated with characteristic LUC activities (Figure 1A). Theoretically, an initial 20-30 recombinants for establishing the physical map and a total of 50-100 recombinants should be sufficient to narrow down the location of the mutation to a 1-4 Mb region [1,3,15]. We suggest that isolation of ~150 or fewer recombinants may sufficient for TPSeq.

After the mutation sites had been narrowed down to three non-overlapping regions of approximately 534 kb, 413 kb and 737 kb, we applied TPSeq (Figure 2A) to reveal the molecular identity of three non-overlapping mutations. The first critical step of TPSeq was to generate high quality mutant libraries within the targeted genome regions by PCR-amplified DNA fragments of average ~7 kb (Additional file 1: Table S1). PCR-primers were designed with an average 200-800 bp overlap with the neighbouring PCR fragment. More than 75% primer pairs worked successfully to cover the targeted regions with the size range of 6-10 kb using routine long-range PCR reactions. For regions that failed to amplify, shorter PCR products (1-6 kb) were redesigned and generated. We covered 99.7% of the sequence in these three mutant regions using this protocol. A total of 75 (*nis1*), 113 (*nis2*), and 138 (*ncr1*) amplicons were generated to cover the targeted regions. After performing PCR, we used agarose gel electrophoresis to confirm and separate non-specific PCR products. This step was important to lower the DNA contamination in the library and to normalize the coverage based on equal DNA molarity. Although not expected for EMS mutagenesis, PCR analysis could potentially reveal insertion, deletion or inversion in the targeted genomic regions. For each mutant, normalized PCR DNA fragments covering the targeted genomic regions were pooled. In order to normalize DNA molarities for each mutant, the pooled PCR mixture from each of the three mutants were combined so that DNA fragments for each mutant was present in equal molarities. The combined DNA fragments were physically sheared to 200 bp, and then ligated to adaptors for NGS in an Illumina HiSeq 2000 genome analyzer.

In our experiment, we covered 99.7% of the genomic sequence in the three targeted mutation regions with 8.5 Gb of sequences generated by NGS (Table 1). In keeping with our intention to make this method accessible to biology laboratories without specialized informatics support, we have composed a detailed bioinformatics analysis workflow that can be performed on the web-based resource Galaxy [16-18]. After uploading a FASTQ file provided by a sequencing facility, all the bioinformatics steps from alignment to SNP (single nucleotide polymorphism) detection can be performed in Galaxy following a simple protocol. This circumvents the need for sophisticated computer hardware and specialized bioinformatic expertise, and makes the bioinformatics analysis of NGS and mutant identification practical and accessible to individual laboratories.

**Table 1:** Sequencing statistics

	Library
**Lane Yield (Mbases)**	8,485
**Read Length**	45
**Clusters (raw)**	4,593,946 ± 382,484
**Clusters (PF)**	3,842,809 ± 305,442
**% PF Clusters**	83.67 ± 0.58
**Total Sequences**	184,454,857
**Sequences Align to Reference**	160,990,234 (87.28%)

PF: Pass Filter

**Figure 3:**
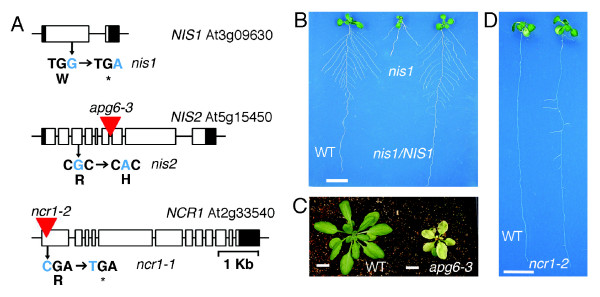
**Confirmation of mutation sites by complementation or analysis of additional allelic mutants**. **A**. Molecular basis of the EMS and insertion mutations. The mutation site in each gene is shown. Red triangle indicates T-DNA insertion site. **B**. Complementation of *nis1 *with the *NIS1 *genomic DNA construct. Plants were grown on medium containing 2.5 mM ammonium succinate for 3 days and transferred to the medium containing 5 mM KNO_3 _for 8 days. **C**. The allelic *apg6-3 *mutant shows similar small pale-green leaves as *nis2*. Plants were germinated on the 1% phyto-agar plates with 1/2 × MS and 1% sucrose for 12 days and then transferred to soil for 22 days. Photograph was taken at day 34 after germination. **D**. The allelic *ncr1-2 *mutant shares similar lateral root de-suppression phenotype as *ncr1-1*. Seedlings were geminated on medium containing 50 mM KNO_3 _for 14 days. Scale bar = 1 cm.

After data analysis, a total of 99.7% of the genomic sequence was covered to a depth of at least one read (Table 2) with only a few small gaps representing AT-rich sequences in the three targeted regions. Considering the coverage rate for the target regions and filtering out the false-positive variants generated by PCR or sequencing, a 20 read depth was set for subsequent analysis. Under this cutoff parameter, a total 98.9% of the targeted genomic sequence was covered (Table 2). In Galaxy, sequences were aligned to the *Arabidopsis *Col-0 genome TAIR10 using Bowtie [19] (Figure 2C). Variants were determined in the web-based resource Galaxy using Samtools pileup [20] and Filter pileup (Table 3). After analyzing, 14 variants were identified and re-confirmed by Sanger sequencing (Table 4 and Figure 3A). Among the remaining true variants, 2 of them are mutated in non-coding region (intron and 3'UTR), 4 of them are within the intergene and 8 of them are exonic. In the 8 exonic variants, 5 of them are missense and 2 of them are nonsense (Table 4). Theoretically, EMS mutagenesis induces a G/C to A/T base transition. In this study, we noticed that 3 confirmed mutations of the total 14 mutations were non-EMS type mutations and they all occurred in *nis1*. We do not know whether these mutations were caused by EMS mutagenesis or another mechanism, but these non-typical EMS-generated mutations have also been observed in other studies where EMS was used [7,9].

**Table 2:** Coverage analysis

	*nis1*	*nis2*	*ncr1*	Total
**1×**	99.36%	99.85%	99.65%	99.67%
**5×**	99.07%	99.44%	99.46%	99.36%
**10×**	99.06%	98.92%	99.44%	99.12%
**15×**	99.05%	98.69%	99.39%	99%
**20×**	99.05%	98.41%	99.32%	98.86%
**100×**	98.72%	90.16%	97.05%	94.44%

**Table 3:** Summary of mutations generated by Galaxy's Filter pileup

**^1^Chr**.	Position	**^2^Ref**. base	**^3^Con**. base	**^4^Con. Qual**.	**SNP Qual**.	**Max**. Mapping **Qual**.	Coverage	^5^QA coverage	Total number of deviants	^6^% deviant reads
II	14,208,479	G	A	225	225	60	3,801	3,407	3,379	99.2
II	14,427,587	G	A	225	225	60	7,847	4,268	4,211	98.7

III	2,849,685	A	C	225	225	60	341	319	316	99.1
III	2,954,586	G	A	225	225	60	7,820	2,236	2,208	98.7
III	3,007,742	C	G	225	225	60	1,438	1,177	1,175	99.8
III	3,113,098	G	A	225	225	60	4,170	3,790	3,776	99.6
III	3,114,003	G	T	225	225	60	937	568	567	99.8
III	3,147,629	G	A	225	225	60	2,539	1,558	1,537	98.7

V	4,851,838	C	T	225	225	60	750	669	668	99.9
V	4,979,060	C	T	202	202	60	172	66	65	98.5
V	4,984,678	C	T	152	152	60	134	18	16	88.9
V	5,016,518	C	T	225	225	60	563	509	504	99
V	5,020,510	C	T	225	225	60	2,961	1,333	1,319	98.9
V	5,355,232	C	T	225	225	60	7,841	6,875	6,821	99.2

1. Chromosome

2. Reference base

3. Consensus base

4. Consensus Quality

5. Quality adjusted coverage

6. The percentage of total number of deviants/quality adjusted coverage

**Table 4:** List of confirmed mutation site

Mutant	Position	Base change	Annotation	Chr
	2,849,685	A→C	intergenic	III
***nis1***	**2,954,586**	**G→A**	**W→Stop**	**III**
	3,007,742	C→G	N→K	III
	3,113,098	G→A	R→H	III
	3,114,003	G→T	D→Y	III
	3,147,629	G→A	L→F	III

	4,851,838	C→T	Q→E	V
	4,979,060	C→T	intergenic	V
	4,984,678	C→T	intergenic	V
***nis2***	**5,016,518**	**C→T**	**R→H**	**V**
	5,020,510	C→T	intron	V
	5,355,232	C→T	3'UTR	V

***ncr1***	**14,208,479**	**G→A**	**R→Stop**	**II**
	14,427,587	G→A	intergenic	II

Chr: Chromosome

### Validation of mutations

We further validated the causal mutations linked to the specific mutant phenotype. Six mutations have been identified in the *nis1 *library based on the *Arabidopsis *Col-0 reference genome TAIR10 (Table 4). Among these mutations, there is only one (G to A) nonsense mutation (Table 3) and this occurs in the first exon of RPL4A (ribosomal protein large subunit 4A, At3g09630) [21] (Figure 3A). To confirm that the altered root architecture is indeed caused by this mutation, the construct containing the genomic DNA fragment of *RPL4A *was shown to complement the *nis1 *root phenotype (Figure 3B). Detailed characterization of the NIS1 functions in nitrate signaling is beyond the scope of this method paper and will be published separately.

In the *nis2 *library, six mutations have been uncovered. One of the mutations (C to T) occurs in the coding region of APG6/CLPB3 (albino or pale-green/casein lytic proteinase B3, At5g15450), which converted a conserved Arg residue to His residue (Table 4). We demonstrated that a T-DNA insertion mutant allele, *apg6-3*, displays the small pale-green leaf phenotype of *nis2-1 *[22] (Figure 3A and Figure 3C). Thus, *NIS2 *encodes APG6 with an important role for nitrate-associated leaf greening and expansion. It has been shown that null *apg6 *mutants cannot survive on soil unless first germinated and grown on medium containing sucrose to bypass certain critical growth points [22]. The *nis2 *mutant has a mis-sense mutation, which can germinate and grow on the soil. It is possible that *nis2 *is a weak mutant caused by the Arg to His substitution and may decrease protein function or activity. It will be interesting to determine how NIS2/APG6 mediate nitrate signaling to control chloroplast development and leaf expansion. There are two mutations revealed by TPSeq in the *ncr1 *library. One candidate shows a G to A substitution causing a stop codon in the C-terminal domain phosphatase-like3 gene (*AtCPL3*, At2g33540). *Arabidopsis *CPL3 is a regulator of stress responsive gene transcription and plant development [23]. We identified an additional T-DNA insertion mutant allele (*ncr1-2*) (Figure 3A), which exhibited similar lateral root elongation as *ncr1-1 *at 50 mM nitrate (Figure 3D). It is possible that *NCR1 *affects expression of genes involved in lateral root elongation through regulation of RNA polymerase II activity. Intriguingly, none of these genes have previously been reported to participate in nitrate signaling. The three novel genes involved in nitrate signaling that were simultaneously uncovered with this method provide a starting point towards elucidating molecular mechanisms underlying these new regulators that will significantly expand our understanding and application of nitrate-associated traits and nitrate signaling networks. Future studies will be required to dissect the complex relationships between nitrate regulation of transcription and growth of different organs

### TPSeq is an efficient and low-cost method

By targeted sequencing of < 1% of the *Arabidopsis *genome for each mutant library, up to dozens of mutants can be pooled for sequencing in one lane and cost is thus minimized. The main expenses of the TPSeq method are accrued in generating the targeted libraries by PCR. A cost assessment analysis showed that amplifying a ~550 kb genomic region by PCR (~7 kb) for mutant identification costs ~500 USD (Additional file 2: Table S2). As DNA synthesis cost has steadily decreased, improving PCR product length (> 10 kb) and reducing volume of the PCR reaction can further lower the cost. Compared to current methods [7-9], which generally cost more than ten thousand USD for the identification of each mutant, TPSeq provides a relatively low-cost strategy for simultaneously identifying multiple mutations. The sequencing data indicated that the accuracy of PCR is not a major concern during genomic DNA amplification and library construction, as we did not detect significant PCR-generated mutations during data analysis. In this study, around 8.5 Gb nucleotide sequences were generated. For each of the targeted regions, the vast majority of the sequences, 98.7% (*NIS1*), 90.2% (*NIS2*) and 97.1% (*NCR1*), were covered at a depth of over 100 (Table 2). This is more than a sufficient read depth to identify the mutation sites based on our 20× reads cutoff. If each mutant could be mapped to a 500 kb size, TPSeq has the potential to simultaneously identify dozens of mutants from one lane of sequencing in an Illumina HiSeq 2000 genome analyzer. In the case where mutation sites of different mutants are located in the overlapping region, sequencing barcodes can be employed to distinguish different mutant libraries [24,25]. Another advantage of TPSeq is that several laboratories with only a few mutants each can combine libraries on a single lane of sequencing making this type of analysis much more feasible and affordable to a greater number of laboratories. In comparison, the NGM approach appeared to carry a higher risk of missing the mutation due to lack of coverage over the target site in WGS [7]. Using a small F2 recombinant population may increase the complexity of validating the causative mutation on the expanded targeted region and confine this method to identifying a single mutant.

We developed a streamlined TPSeq method and combined it with powerful genetic screens in an experiment to simultaneously identify three novel nitrate-signaling mutants. In doing so, we demonstrate the potential of this method for simultaneously identifying dozens of mutants at low-cost and thus enabling it to more fully exploit the information generated by genetic screens essential for dissecting complex signaling networks. Importantly, we ensure that the necessary bioinformatics processing is accessible to laboratories without specialized computational hardware and personnel by providing a straightforward protocol for executing all of the NGS data analysis on the web-based Galaxy. This means that plant laboratories geared towards isolating and mapping multiple mutants but without specialized resources to identify them can greatly benefit from TPSeq. The method enables the amount of information gained in NGS to be more commensurate with ambitious genetic screens and as a consequence greatly increases the power of discovery.

## Conclusions

We have demonstrated that TPSeq is a practical and economical method for every laboratory to fully realize the advantage and promise of forward genetic screens in unraveling the molecular basis of complex signaling networks in *Arabidopsis *and other genetic model systems with complete genome sequences. It has the potential to simultaneously identify dozens of mutants using a single lane of sequencing based on the performance of the Illumina HiSeq 2000 platform using single-end reads of 45 cycles and generating approximately 184 million reads. We validated and confirmed the molecular mutations causing the nitrate-associated mutant phenotypes by either genetic complementation or by analyzing additional mutant alleles. TPSeq can be especially advantageous when applied to genetic model systems with large sequenced genomes such as maize or mouse, as targeted sequencing of only genetically-defined genomic regions significantly reduces costs and efforts in identifying mutations.

## Materials and methods

### Plasmid construction and the generation of transgenic plants

The 2.5 kb NIR promoter was amplified from *Arabidopsis *genomic DNA with two primers, NIR-F: 5'-GGGGGATCCTAAGAAGTAAGAACGGTGAT-3' and NIR-R: 5'- GGGCCATGGGATGATGGCGGAAGAAGG-3'. The amplified DNA was then fused to the luciferase (LUC) reporter to generate a *NIR-LUC *construct. In order to generate a *NIR-LUC *transgenic line, *NIR-LUC *was cloned into the binary vector, pBIN19, and plant transformation was accomplished with the floral-dip method [26]. The *Arabidopsis *lines harboring a single copy of the T-DNA insert were selected based upon kanamycin resistance in the T2 generation and copy number was then determined by performing Southern blot analysis using the coding region of *NPTII *as a probe. One *NIR-LUC *transgenic line was selected for subsequent study based on its showing a higher LUC activity in response to nitrate induction.

### EMS mutagenesis

Approximately 60,000 seeds from the *NIR-LUC *homozygous transgenic line were treated with 0.2% ethyl methanesulfonate (EMS) for 16 h at 24°C in the dark. Mutagenized seeds were planted and M2 seeds were produced and pooled for screening.

### Two-step mutant screen

The first step of the *nis *&*ncr *mutant screen consisted of growing *NIR-LUC *transgenic wild type seedlings in a 96-well plate and then using a scintillation counter (PerkinElmer) to detect and compare LUC activity in order to identify *nis *and *ncr *mutants. Briefly, 200 μl of the basal medium [27] (10 mM KH_2_PO_4_/KH_2_PO_4 _pH 5.5, 1 mM MgSO_4_, 1 mM CaCl_2_, 0.1 mM FeSO_4_-EDTA, 50 μM H_3_BO_4_, 12 μM MnSO_4_·H_2_O, 1 μM ZnCl_2_, 1 μM CuSO_4_·5H_2_O, 0.2 μM Na_2_MoO_4_·2H_2_O, 1 g/L MES, and 0.5% sucrose, pH 5.8) with 0.8% phytoagar and 2.5 mM ammonium succinate as the sole nitrogen source was loaded into each well. A single seed was then germinated in each well under constant light, at 25°C for 6 days. To screen for *ncr *mutants, a total volume of ~0.5 ml (0.1 ml/spray) of a 0.5 mM luciferin and 0.1% Triton-X-100 solution was sprayed on each plate with a plastic fine mist sprayer, and then the plate was kept in darkness for 5 minutes before being placed into a scintillation counter. Luminescence counts from LUC activity was measured in counts per 5 seconds. Seedlings with higher counts than wild type were selected and propagated. The results were re-confirmed using the same procedure on seedlings from the second generation. Seedlings remaining on the plate were maintained under the same growth conditions for an additional day. Screening for *nis *mutant seedlings was accomplished by adding 10 μl of 200 mM KNO_3 _to each well for 2 h. The plate was then sprayed with luciferin solution, kept in darkness for 5 minutes and placed into the scintillation counter as described above. In this case, seedlings with counts lower than wild-type seedlings, *nis1 *plants, were selected. Selected mutants were propagated and the results were confirmed in second generation seedlings. Approximately 25,000 M2 seedlings were screened. A total of 273 *nis *mutants and 65 *ncr *mutants were isolated during the first step of the screen. Of these, 4 *nis *and 5 *ncr *mutants were confirmed in the second generation. These were then subjected to a secondary screen. Secondary screens on *nis *or *ncr *mutants were performed to identify defects in three nitrate-associated traits; retarded lateral root elongation, small pale green leaves, and liberation of high-nitrate inhibition of lateral root elongation. In order to screen for mutants defective in nitrate-induced lateral root elongation, seedlings were germinated on a 2.5 mM ammonium succinate medium (describe above) with 1% phytoagar under constant light (150 μE) at 22°C for 3 days. Plants were then transferred to a 5 mM KNO_3 _medium for 8 days, and the mutants displaying a shorter lateral root in comparison to wild type were selected. Screening of mutants for the high-nitrate inhibition of lateral root elongation trait was accomplished by first growing plants on a 10 × 10 cm square plate in a 50 mM KNO_3 _medium (describe above) with 1% phytoagar under a 12 h light (100 μE) 23°C/12 h dark 20°C regime for 14 days. Plants showing a readily visible lateral root (> 0.5 mm) were selected. Mutants with the small pale green leaf trait were visually identified after growing in soil under a 12 h light (100 μE, 23°C)/12 h dark (20°C) regime for 33 days.

### Genetic and physical mapping of mutants

To obtain a transgenic line containing a *NIR-LUC *construct in the L*er *ecotype, a Col-0 plant containing the *NIR-LUC *construct was backcrossed to wild type L*er *plants for one generation and the progeny L*er *were backcrossed to self for five generations. Kanamycin resistance was used as a marker to select for the presence of the *NIR-LUC *transgene. Ten PCR-based molecular markers (ADH1, NGA62, C4H, ER, CA1, BGL1, DET1, NGA1107, CA72, and ATTED2; http://www.arabidopsis.org/servlets/Search?action=new_search&type=marker), each located near the upper and lower arm of a chromosome, were used to identify the L*er *ecotype. One transgenic line containing 9 markers associated with the L*er *ecotype and one Col marker (DET1) on Chromosome IV (*NIR-LUC *was inserted on Chromosome IV upper arm) was chosen for mutant mapping.

Plants previously identified as *nis1, nis2 *and *ncr1 *mutants were crossed to this *NIR-LUC*/L*er *line and recombinant containing plants were identified by monitoring LUC activity. Quick genetic mapping was then performed on the F2 population. Genomic DNA was extracted from recombinant plants. Simple sequence length polymorphism (SSLP) or cleaved amplified polymorphism sequences (CAPs) markers were used for PCR-based genotyping to narrow down the mutation location [1]. Fine mapping between adjacent markers shown in Figure 2B was accomplished with information from the TAIR website http://www.arabidopsis.org/servlets/Search?action=new_search&type=marker, and new CAPs and SSLP markers were designed using SNP and INDEL information available from the TAIR Polymorphisms/Alleles database http://www.arabidopsis.org/servlets/Search?action=new_search&type=polyallele. The CAPs (restrict enzyme name, Col/L*er*, in bp) and SSLP (Col/L*er*, in bp) markers shown in Figure 2B are listed below:

*NIS1*: F3L24-1, 5'-TGCCTGTTTGCTTCATTCTG-3', and 5'-CGCAAAACTGCAAAGTACA-3' (*Bcl*I, 442/357 + 85); SGCSNP6754, 5'-CAGAGAACCTTTCTGTTGCAC-3', and 5'-GATGCAACTCCTGTGCTCAA-3' (*Mse*I, 30 + 179/30 + 82 + 97).

*NIS2*: MQK4-1, 5'-AGGTCACGATTGTTTCTTTGC-3', and 5'-GGTCCTTCAATAAACTTCAA-3' (*Cla*I, 549/312 + 237); NGA151, 5'-CAGTCTAAAAGCGAGAGTATGATG-3' and 5'-GTTTTGGGAAGTTTTGCTGG-3' (150/120).

*NCR1*: F24L7-1, 5'-GATTCAGATTGGGGAAGCAA -3', and 5'-CTGCAATGTCAAACGCATCT-3' (*ClaI*, 383 + 287/383 + 172 + 115); F13P17-1, 5'-CCCGGTCACCTAACTTACCA-3' and 5'-GAGCCCAAGCCCATTAGACT-3' (198/206).

### LUC activity assay

Plants were grown under constant light (150 μE) for 7 days on 10 × 10 cm square plates with 30 ml medium, 1% phytoagar at pH 5.8, and 2.5 mM ammonium succinate as the sole nitrogen source. The nitrate induction was determined by adding 10 ml of 10 mM KNO_3 _or 10 mM KCl to the plate for 2 h. Three seedlings were pooled into one tube, then ground into fine powder in liquid nitrogen and resuspended in 50 μl cell lysis buffer (25 mM Tris-phosphate, pH 7.8, 2 mM DTT, 2 mM 1,2-diaminocyclohexane-N, N, N', N'-tetraacetoc acid, 10% glycerol and 1% Trixon X-100). Supernatant (20 μl) was taken for the LUC assay (Luciferase assay system, Promega) [28]. Scintillation counting (PerkinElmer) was used to measure LUC activity. Protein concentration was determined with the Biorad protein assay system (Biorad).

### RNA isolation and real-time RT-PCR

Approximately 10 seedlings were pooled and total RNA was isolated using Trizol reagent (Invitrogen). First strand cDNA was synthesized from 1 μg of total RNA using the Imporm-II reverse transcription system (Promega) in a total volume of 20 μl. Real-time RT-PCR was carried out by iCycler iQ real-time PCR-detection system using iQ SYBR green supermix (Biorad). For each PCR reaction, 0.5 μl of the reverse transcription reaction was used. The primers used were: *NIR*, NIR-RT-F: 5'-GACGAACTTGGTGTTGAAGG-3' and NIR-RT-R: 5'- TGTAGCCTACCAACCGGAAC-3'. *TUB4*, TUB4-F: 5'-CGAAAACGCTGACGAGTGTA-3' and TUB4-R: 5'-GAAGTGAAGCCTTGGGAATG-3'.

### Complementation and isolation of allelic mutants

Complementation analysis in *nis1 *was performed by first amplifying a 3 kb DNA fragment from wild type Col-0 *Arabidopsis *genomic DNA using two primers, (At3g09630-F: 5'-cgggatccaacgcaacaaatcccgatag-3'; At3g09630-R: 5'-gctctagacgagcacaaaaacgttaggg-3'). The amplified DNA fragment was then digested with *Bam*HI and *Xba*I and cloned into the binary vector pCB302 [29]. The *nis1 *mutant was complemented with this construct using *Agrobacterium*-mediated transformation.

Two T-DNA insertion allelic mutants, *apg6-3 *(Salk_071039) and *ncr1-2 *(Salk_143411), were obtained from the Arabidopsis Biological Resource Center (ABRC) [30]. Homozygous T-DNA insertion lines were identified by PCR using specific *APG6 *and *NCR1 *gene primers and a T-DNA left border primer. The primers are listed below: LBa1, 5'- TGGTTCACGTAGTGGGCCATCG-3'; *APG6*, Forward 5'-

GGCCACTGATGTAACGGTCT-3', and reverse 5'- GATAAGCGGTTTGGGAAACA-3';

*NCR1*, Forward: 5'-GTTTCTGAATCGGGTTTGGA -3', and Reverse: 5'- CGCTGAAACGAAACAGAACA-3'

The pale-green phenotype exhibited in *apg6-3 *plants was selected for by first germinating seeds on a 10 × 10 cm square plate with 30 ml of medium containing 1/2 × MS, 1% sucrose, 0.8% phytoagar, and 1 g/l MES pH 5.8 for 12 days. Plants were then transferred to soil under the same condition as the *nis2 *experiment for 22 days. On day 34, plants were photographed.

### TPSeq

#### Genomic DNA isolation

Approximately 100 *Arabidopsis *seedlings were grown on 1% phyto-agar (Plantmedia) plates with 1/2 × MS and 1% sucrose, pH 5.8 under constant light (75 μE) at 22°C for 5 days. Seedlings (approximately 0.6 g fresh weight) were ground to a fine powder in liquid N_2_, and genomic DNA was isolated in accord with the CTAB DNA extraction protocol [31]. The concentration of total nucleic acid was measured with a NanoDrop (NanoDrop 1000; Thermofisher Scientific). The Genomic DNA isolated using the CTAB protocol contained RNA, and although RNase treatment is not necessary for generating the libraries described in the TPSeq protocol, the concentration of genomic DNA without RNase treatment was ~6.5 × higher than that with RNase treatment (i. e., 6.5 ng total nucleic acid isolated by CTAB method = 1 ng genomic DNA).

#### Primer design and PCR

The genomic sequence (300-700 kb) between marker genes for each mapped mutant was downloaded from TAIR http://www.arabidopsis.org/, and used with online software, Primer3 http://frodo.wi.mit.edu/primer3/, for designing primers. Primers were designed for every 6-10 kb or for a shorter fragment size (1-6 kb) of overlapping genomic DNA using the default settings on the Primer3 website (Additional file 1: Table S1). Primers were designed with an average 200-800 bp PCR fragment overlap with the neighbour PCR fragment. Three reaction mixtures were used for amplifying PCR products. Reaction mixture 1 was used for most PCR amplification, but if no PCR product was amplified using reaction mixture 1, then reaction mixture 2 or reaction mixture 3 was used. If PCR product could not be obtained with any of the three reaction mixture then primers generating a shorter fragment size (1-6 kb) were designed and used. Three reaction mixtures were prepared as below: Reaction mixture 1: Reaction mixture contained 24 ng *Arabidopsis *genomic DNA, 1 μl 10 × #2 expand long template buffer, 300 μM dNTP mix, 400 nM mixed primer pair, 1% DMSO and 0.6 unit (5 U/μl) expand long template enzyme mix (Roche) in a final volume of 10 μl; Reaction mixture 2: This PCR reaction mixture contained the same as condition 1, except an additional 1 mM MgCl_2 _(final 3.75 mM) was added to the reaction in a final volume of 10 μl; Reaction mixture 3: Reaction mixture was the same as 1 except 1 μl 10 × #1 expand long template buffer was used.

PCR was then performed with a C1000 Thermal cycler (BioRad) according to the following protocol: 3 min at 95°C, 10 sec at 95°C, 15 sec at 55°C, 8 min at 68°C (repeat 35 cycles), and 10 min at 68°C. After performing PCR, the PCR product was checked on a 0.8% agarose gel. Approximately 150 ng of amplified DNA (adjusted to roughly equal molar amounts as estimated from DNA intensity in the 0.8% gel) was loaded onto and separated in a 0.6% agarose gel. For each mutant, DNA bands were cut and pooled for purification using a gel purification kit (Qiagen), and each pooled DNA concentration was measured using a NanoDrop.

#### DNA shearing

The volume of PCR mixtures for each mutant was adjusted so that all three mixtures had approximately equal DNA molarities (NCR1 [534 kb]: 1.65 μg, NIS1[413 kb]: 1.2 μg, and NIS2 [737 kb]: 2.15 μg). The three mixtures were then pooled into one tube. A 100 μl aliquot of this pooled mixture (5 μg) was subjected to acoustical fragmentation of DNA with a Covaris S2 adaptive focused acoustics disruptor (Covaris). DNA fragments of approximately 200 bp were obtained with the following settings: intensity 5, duty cycle 5%, bust 200/sec, and mode frequency sweeping for 15 min.

#### DNA end repair

Reactions were carried out in a PCR tube containing 2.5 μg DNA fragment, 1 × T4 DNA polynucleotide kinase buffer (NEB), 100 μM dNTP mixture, 40 U T4 polynucleotide kinase (NEB), 5 U Klenow (NEB), 1 mM ATP and 12 U T4 DNA polymerase (NEB) in a final volume of 100 μl. PCR tube was incubated in a thermal cycler (BioRad) at 20°C for 1 h and enzyme was then inactivated by raising the temperature to 65°C for 30 min.

#### A-tailing

Klenow 3' → 5' exo polymerase (5 U, NEB) and 4 μl of 100 mM dATP were added to the 100 μl reaction after performing DNA end repair. The mixture was incubated in 37°C for 50 min. Repaired and A-tailed DNA fragments were purified with a Qiagen PCR purification kit (Qiagen). The mixture of purified DNA fragments was then concentrated to 40 μl with a Thermal savant speed vacuum (Thermal Scientific), and the concentration of DNA was determined with a NanoDrop.

#### Adaptor ligation

Two oligos were synthesized and HPLC purified by Sigma-Aldrich:

Adaptor 1: 5'-AATGATACGGCGACCACCGAGATCTACACTCTTTCCCTACACGACGCTCTTCCGATC*T-3'. * indicates phosphorothioate

Adaptor 2: 5'-GATCGGAAGAGCGGTTCAGCAGGAATGCCGAGACCGATCTCGTATGCCGTCTTCTGCTTG-3'

The adaptors were phosphorylated at the 5' end in a reaction mixture composed of 40 μM of each adaptor, 20 U T4 polynucleotide kinase (NEB), 1 × T4 polynucleotide kinase buffer (NEB) and 1 mM ATP in a total volume of 100 μl in a PCR tube incubated in a water bath at 37°C for 30 min. The tube containing the reaction mixture was then placed in boiling water for denaturing and cooled to room temperature for annealing.

The ligation reaction was carried out in PCR tube in a total volume of 50 μl containing 0.5 μg end repaired and A-tailed DNA, 2 μM adaptor mixture from above, 1 × T4 Quick T4 DNA ligation buffer (NEB), 5 μl of Quick T4 DNA ligase (NEB). The PCR tube was incubated for 30 min at 20°C in a thermal cycler (BioRad).

#### Size selection

The ligated library was separated on a 2% agarose gel, and fragments between 250 and 350 bp were eluted and purified by gel extraction (Qiagen). The library was then dissolved in 80 μl H_2_O.

#### Library amplification

The library amplification reaction containing 10 μl ligated DNA library, 1 × Phusion buffer (NEB), 250 μM of dNTP mix, 0.5 U Phusion high fidelity DNA polymerase (NEB), and 50 nM of each primer in total volume of 50 μl: Library-F: 5'-AATGATACGGCGACCACCGAGATCTACACTCTTTCCCTACACGA-3'. Library-R: 5'-CAAGCAGAAGACGGCATACGAGATCGGTCTCGGCATTCCTGCTGAAC-3' The PCR protocol consisted of 3 min at 98°C, 15 sec at 98°C, 15 sec at 65°C, 30 sec at 72°C (repeat 10 cycles), and 5 min at 72°C and was run on a thermal cycler (Biorad). PCR product was separated on a 2% agarose gel and the size between 180-300 bp was eluted and purified with a gel extraction kit (Qiagen). An Agilent Bioanalyzer was used to determine quality and quantity of the DNA library.

#### Next generation sequencing

The DNA library was concentrated to 1.8 nM, and TPSeq on single end reads was done for 45 cycles on an Illumina HiSeq 2000 using one lane of a flow cell. After sequencing, the sequencing facility (Biopolymers laboratory, MIT, USA) provided us with a FASTQ file.

#### Data analysis

We used the web-based tool Galaxy [16-18] to analyze data. The FASTQ file provided by our sequencing facility was uploaded to the Galaxy website http://main.g2.bx.psu.edu.

A quality check of the sequencing was assessed using FastQC which is found under the NGS: QC and manipulation heading in the NGS Toolbox section of Galaxy. SNP analysis was performed in accord with the following procedure:

1. The uploaded FASTQ file was first re-formatted using FASTQ Groomer. This step replaces Illumina coded quality scoring in the FASTQ file to Sanger code quality scores and allows for subsequent analysis with Galaxy. FASTQ Groomer is located under the NGS: QC and manipulation header in Galaxy. FASTQ files from Illumina 1.8 now use Sanger code quality scores instead of Illumina coded quality scores and with these files this step is unnecessary. Note: Large files, > 10 GB, may take several days to run the FASTQ Groomer. Splitting the FASTQ file into several files and running multiple instances of FASTQ Groomer in parallel greatly speeds up this step to a matter of hours. The FASTQ file can be split using a perl script.

2. The resulting Sanger formatted file was then used as input into 'Map with Bowtie for Illumina (under the NGS: Mapping header in Galaxy). Sequences were mapped to the *Arabidopsis *TAIR 10 reference genome using the default settings found in Galaxy.

3. The alignment file resulting from step 2 is in SAM format and needs to be converted to a binary, BAM, format before being used as input into pileup. The SAM format alignment file was converted to BAM format with 'SAM-to-BAM' located under the NGS: SAM Tools [20] heading in Galaxy.

4. The BAM file was used as input into 'Generate pileup' also found under the NGS: SAM Tools header. Default parameters were used with two exceptions: 'print the mapping quality as the last column' was chosen in the pull-down menus and the consensus was called according to the MAQ model.

5. The file produced from running 'Generate pileup' was then used as input into the 'Filter pileup' function. Default parameters were used with three exceptions: 'Do not report positions with coverage lower than' was set to 20, and 'Print total number of differences' and 'Pileup with ten columns (with consensus)' were chosen.

6. The resulting file was filtered in Galaxy with the 'Filter' function found under 'Filter and Sort' heading. In the 'With following condition' box was inserted 'c3! = c4 (The preceding should read 'c3! = c4')'. This filtered out all lines in which the reference base was the same as the consensus base.

7. Using 'Compute' found under the 'Text Manipulation' heading the above created file had an additional column added in which the percentage of variants per quality adjusted coverage was calculated by inserting '(c17/c16)*100' in the 'Add expression' box.

8. The resulting file was downloaded from Galaxy and imported into Excel and Excel's Filter function was used to show variants in target regions.

For determination of coverage and visualization of mutations, the alignment file generated in Galaxy in SAM format (the output file resulting from bowtie alignment) was sorted in order to facilitate being loaded into Tablet after being downloaded from Galaxy. After choosing 'Sort' located under the 'Filter and Sort' heading in the left side menu, 'on column' was changed to 'c3', 'with flavor:' was changed to 'Alphabetical sort', and 'everything in:' was changed to 'Ascending order'. The 'Add new Column selection' was chosen and in this new menu under the header 'Column selection 1', 'on column' was changed to c4, 'with flavor' was kept at the default, and 'everything in' was changed to 'ascending order'. This will sort the SAM alignment file according to chromosome (c3) and position on chromosome (c4).

The sorted file was downloaded from Galaxy and imported into Tablet NGS assembly visualization software [32]. A plain text summary coverage file was then exported from Tablet, and information in this file was processed with perl scripts. One script was used to calculate the average coverage over a given genomic region. The output of this script was imported into Excel in order to make chromosome coverage graphs. Other perl scripts were used to calculate coverage for each base as well as an average coverage for specified genome regions. In the present report, these were calculated on chromosome II between base positions 13,899,498-14,434,486, chromosome III between base positions 2,825,193-3,234,290, and chromosome V between base positions 4,668,273-5,401,599. All perl scripts are available on request.

## Competing interests

The authors declare that they have no competing interests.

## Authors' contributions

K-HL and JS initiated the project and designed the experiments; K-HL carried out the experiments; MM and K-HL analyzed sequencing data; K-HL, MM and JS wrote the manuscript. All authors read and approved the final manuscript.

## Supplementary Material

Additional file 1 **Table S1. Primer sequences for PCR libraries**. Click here for file

Additional file 2 **Table S2. Cost assessment for TPSeq**. Click here for file
