# Supplementary material for: Targeted parallel sequencing of large genetically-defined genomic regions for identifying mutations in Arabidopsis
Source: Plant Methods. 2012 Mar 30;8:12. doi: 10.1186/1746-4811-8-12 (PMC3348062; doi:10.1186/1746-4811-8-12)
Supplement: Additional file 2 — Table S2. Cost assessment for TPSeq. [file 1746-4811-8-12-S2.PDF]

Table S2. Cost assessment for TPSeq

Step	Category	Description	Cost per three mutants (USD)[1]	Cost per mutant[2] (USD)
**Amplify targeted genomic DNA**	Oligonucleotides synthesis	326 primer pairs, average 20 bases each (0.1 USD / base)	1304	434
	Taq polymerase	326 reactions, 0.6 U / reaction	129	43
	96-well PCR plate	4 plates	12	4
	Gel purification	6 Qiaquick gel extraction columns and ~ 100 g low melting agarose	60	20
		Total	1505	**501**
**Library construction**	Enzymes	T4 DNA polymerase, Klenow 3'→5' exo polymerase, Klenow, T4 polynucleotide kinase, Quick T4 DNA ligase, and Phusion polymerase	20	
	Adaptor	Phosphorothioate modification & HPLC purification	85	
	Purification	Gel purification and Qiagene column	10	
	Bioanalyzer analysis	Chip and reagent kit	55	
		Total	**170**[3]	

1. US dollars
2. The average cost for identifying a mutant with an average of a size ~550 kb.
3. The cost of library construction for pooled PCR from multiple mutants.
